# Drug-class-specific changes in the volume and cost of antidiabetic medications in Poland between 2012 and 2015

**DOI:** 10.1371/journal.pone.0178764

**Published:** 2017-06-05

**Authors:** Andrzej Śliwczyński, Melania Brzozowska, Andrzej Jacyna, Petre Iltchev, Tymoteusz Iwańczuk, Waldemar Wierzba, Michał Marczak, Katarzyna Orlewska, Piotr Szymański, Ewa Orlewska

**Affiliations:** 1 Division of Quality Services, Procedures and Medical Standards, Medical University in Lodz, Lodz, Poland; 2 National Health Fund, Warsaw, Poland; 3 Health Care Policy Department, Medical University of Lodz, Lodz, Poland; 4 The Agency for Health Technology Assessment and Tariff System, Warsaw, Poland; 5 University of Humanities and Economics, Lodz, Poland; 6 Medical University of Warsaw, Warsaw, Poland; 7 Institute of Cardiology, Warsaw, Poland; 8 Jan Kochanowski University in Kielce, Kielce, Poland; Medical University of Vienna, AUSTRIA

## Abstract

**Aim:**

to investigate the drug-class-specific changes in the volume and cost of antidiabetic medications in Poland in 2012–2015.

**Methods:**

This retrospective analysis was conducted based on the National Health Fund database covering an entire Polish population. The volume of antidiabetic medications is reported according to ATC/DDD methodology, costs—in current international dollars, based on purchasing power parity.

**Results:**

During a 4-year observational period the number of patients, consumption of antidiabetic drugs and costs increased by 17%, 21% and 20%, respectively. Biguanides are the basic diabetes medication with a 39% market share. The insulin market is still dominated by human insulins, new antidiabetics (incretins, thiazolidinediones) are practically absent. Insulins had the largest share in diabetes medications expenditures (67% in 2015). The increase in antidiabetic medications costs over the analysed period of time was mainly caused by the increased use of insulin analogues.

**Conclusions:**

The observed tendencies correspond to the evidence-based HTA recommendations. The reimbursement status, the ratio of cost to clinical outcomes and data on the long-term safety have a deciding impact on how a drug is used.

## Introduction

Diabetes mellitus is a prevalent multisystem metabolic disease associated with high health care resource expenditures. During the last decade, we have witnessed the development of several new antidiabetic medications, e.g. the dipeptidyl peptidase-4 (DPP-4) inhibitors and glucagon-like peptide 1 (GLP-1) agonists, as well as a restriction of thiazolidinediones use as a result of safety monitoring data [1]. Effectiveness of treatment is often influenced by selection of medicines, therapy changes as well as patient adherence with prescribed drug regimens. In every country a large number of socio-cultural factors contribute to the ways drugs are used, e.g.: national drug policy, drug advertising and promotion, availability of independent drug information, international and local clinical guidelines. Drug utilization studies which are defined as the marketing, distribution, prescription and use of drugs in a society with special emphasis on the resulting medical, social and economic consequences [2], provide useful insights into the current prescribing practices in typical usual-care settings and play a significant role in helping the health-care system to understand, interpret and improve the prescribing administration and use of medications. Administrative databases seem to be useful in analysing trends in the prescription pattern and the adherence to the cost-effective care recommended by both international and local guidelines [3]. The aim of our study was to investigate the drug-class-specific changes in the volume and cost of antidiabetic medications in Poland during years 2012–2015 and to assess how the observed tendencies correspond to the evidence-based HTA recommendations

## Materials and methods

This is retrospective observational study based on healthcare administrative data from the National Health Fund (NHF), covering an entire Polish population of about 38 million inhabitants. NHF database is the largest database to collect information on patient demographics, disease diagnosis, management and prescribing from Polish out-patient and in-patient care. Every health care institution in Poland is obliged to collect the data on each patients’ primary health care visit, specialist consultations and hospitalisations and refer them to the NHF [4, 5]. Information in the NHF database is updated simultaneously with patient care and includes: patient identification code, diagnose code, medical service code, drug code, dose, formulation, number of packages, drug price, dispensing date, and the health unit where the drug was dispensed. From this database we extracted data on the number of patients receiving at least one prescription of any antidiabetic medications and data about consumption and cost of antidiabetic medications for each year during a study period (January, 1 2012 to December, 31 2015). The volume and cost data were based on dispensed products. Permission to use this database in our study was granted by the responsible authority. Because the data we used was anonymous, neither ethical committee approval nor informed consent was required.

We have grouped the antidiabetic medications according to the Anatomical Therapeutic Chemical (ATC) classification system into following categories: insulins (ATC:A10A), metformin and other biguanides (ATC:A10BA), sulfonylureas (ATC:A10BB), alpha-glucosidase inhibitors (ATC: A10BF), thiazolidinediones (ATC:A10BG), and DPP-4 (ATC:A10BH). The amounts of antidiabetic drugs consumed was measured using defined daily dose (DDD) methodology, which is the standard method recommended by the WHO for drugs utilization studies [2]. To calculate the number of DDDs used, the amount of active substance, for each ATC code, expressed in physical units was calculated and then divided by the DDD associated with this active substance, expressed in the same unit. Standard DDD was obtained from WHO Collaborating Centre for Drug Statistics Methodology (ATC/DDD index 2017) [6]. The data about the consumption of medicines were expressed as DDDs and DDDs/ 1000 inhabitants/day (DDD/TID)

The yearly cost of drugs was calculated by multiplying the number of packages prescribed during the year by the unit cost at the time of prescription. Costs include public payer and patient payment (drug retail price = reimbursement + patient’s co-payment) and are reported in current international dollars, based on purchasing power parity (in 2015 1 USD = 1.776 Polish zloty), which enabled omitting the values of inflation and exchange rate variations in the analysis. [7] The data about cost of medicines were also expressed as USD/ 1000 inhabitants/day (USD/TID).

We calculated the percentage proportions of antidiabetic drugs utilization within total drug utilization and the percentage changes in antidiabetic drugs utilization during the 4-year period.

A descriptive statistics was used to report all data on drugs consumption and costs. The results were presented in absolute numbers, frequency (in %) and graphically. All calculations were performed using Microsoft Excel 2016 software.

## Results

### Diabetes mellitus morbidity trends

During a 4-year observational period the number of patients receiving at least one prescription of any antidiabetic medications increased by 17% from 2,109,263 (54.7 per 1000 inhabitants) in 2012 to 2,462,564 (64 per 1000 inhabitants) in 2015. In the consecutive years, the number of diabetic patients rose annually by 6% (in 2013) and 5% (in 2014, 2015). The increase in 353,301 diagnoses over 4-year period included 88,325 newly diagnosed (registered) diabetic patients per year. According to this figure the observed incidence of diabetes mellitus in Poland is 2.3 patients per 1000 inhabitants per year.

### Antidiabetic drug utilization and costs

The annual volume of antidiabetic medications dispensed in years 2012–2015 in Poland is presented in Table 1. Compared to 2012, the number of issued DDDs showed a relative increase of medication consumption: by 10% in 2013, by 9% in 2014 and by 1% in 2015. As a consequence, in 2015 the number of dispensed DDDs was 21% higher than in 2012. 72% of total consumption was of oral antidiabetics. The two most frequently dispensed subgroups to treat diabetes in Poland were sulfonylureas, with shares varying from 38% (in 2012) to 33% (in 2015), and biguanides, with shares varying from 33% (in 2012) to 39% (in 2015). In 2015 metformin was the most widely used antidiabetic drug with a constant increase during the observation period, from 16.86 DDD/TID in 2012 to 24.32 DDD/TID in 2015 (total increase of 44%). The consumption of sulfonylureas was 19.81 and 20.45 DDD/TID in 2012 and 2015, respectively and there was a relative 7% decrease in the use of sulfonylureas in 2015 in comparison to 2014. The utilization of alpha-glucosidase inhibitors dropped by 10%. DPP-4 inhibitors were less utilised but with constant increase from 0.0015 DDD/TID in 2012 to 0.0057 DDD/TID in 2015. Thiazolidinediones were practically absent on the Polish market. As a class, insulin use has remained relatively stable, accounting for 28% of total antidiabetic drug consumption in Poland. The consumption of all insulins was 14.27 and 17.46 DDD/TID in 2012 and 2015 (increase by 22%).

**Table 1:** Volume and cost of antidiabetic medications in Poland. Number of DDDs in the thousands. Costs in USD x 1000.

ATC	Drug class		2012	2013	2014	2015
A10A	Insulins	Volume; DDD (%)	201,263 (28)	226,851 (28)	239,288 (27)	244,988 (28)
		Cost; USD (%)	356,707 (65.68)	402,819 (66.87)	424,599 (69.21)	436,645 (66.77)
A10BA	Metformin	Volume; DDD (%)	237,769 (33)	273,440 (34)	312,224 (36)	341,239 (39)
		Cost; USD (%)	89,283 (16.44)	102,821 (17.07)	99,910 (16.28)	124,906 (19.10)
A10BB	Sulfonylureas	Volume; DDD (%)	279,367 (38)	291,672 (36)	309,439 (36)	286,947 (33)
		Cost; USD (%)	85,319 (15.71)	85,610 (14.21)	78,483 (12.79)	82,143 (12.56)
A10BF	Alpha glucosidase inhibitors	Volume; DDD (%)	9,853 (1)	9,757 (1)	9,496 (1)	8,845 (1)
		Cost; USD (%)	11,654 (2.15)	10,983 (1.82)	10,296 (1.68)	9,921 (1.52)
A10BG	Thiazolidinediones	Volume; DDD (%)	0 (0.00)	0 (0.00)	0 (0.00)	0 (0.00)
		Cost; USD (%)	0 (0.00)	0 (0.00)	0 (0.00)	0 (0.00)
A10BH	DPP4 inhibitors	Volume; DDD (%)	21 (0.003)	32 (0.004)	57 (0.007)	81 (0.009)
		Cost; USD (%)	114 (0,02)	165 (0.03)	246 (0.04)	318 (0.05)
Total		Volume; DDD (%)	728,273 (100.00)	801,758 (100.00)	870,504 (100.00)	882,100 (100.00)
		Cost; USD (%)	543,077 (100.00)	602,398 (100.00)	613,534 (100.00)	653,933 (100.00)

DDD: definded daily dose; DPP-4 inhibitors: dipeptidyl peptidase-4 inhibitors; USD:

Between 2012 and 2015 the cost of antidiabetic medications has increased by 20% (from USD 543,077,000 to USD 653,933,000) (Table 1). The highest relative increase of costs compared to the preceding year occurred in 2013 (by 11%), followed by a 2% and 7% rise in the subsequent years. Spending on insulins was greater than the combined spending on oral antidiabetic medication (67% vs. 33% in 2015). Following insulins usage trend, the total spending on insulin increased by 22% compared to 2012 (the annual growth rates being 13% in 2013, 5% in 2014 and 3% in 2015). The rate of increase in costs of oral antidiabetics was 16% but changes in individual subgroups were markedly different. The increase was observed in DPP4 inhibitors (by 179%) and metformin (by 40%), while the costs of sulfonylureas decreased by 4%, and the costs of alpha glucosidase inhibitors have been reduced by 15%.

**Fig 1:**
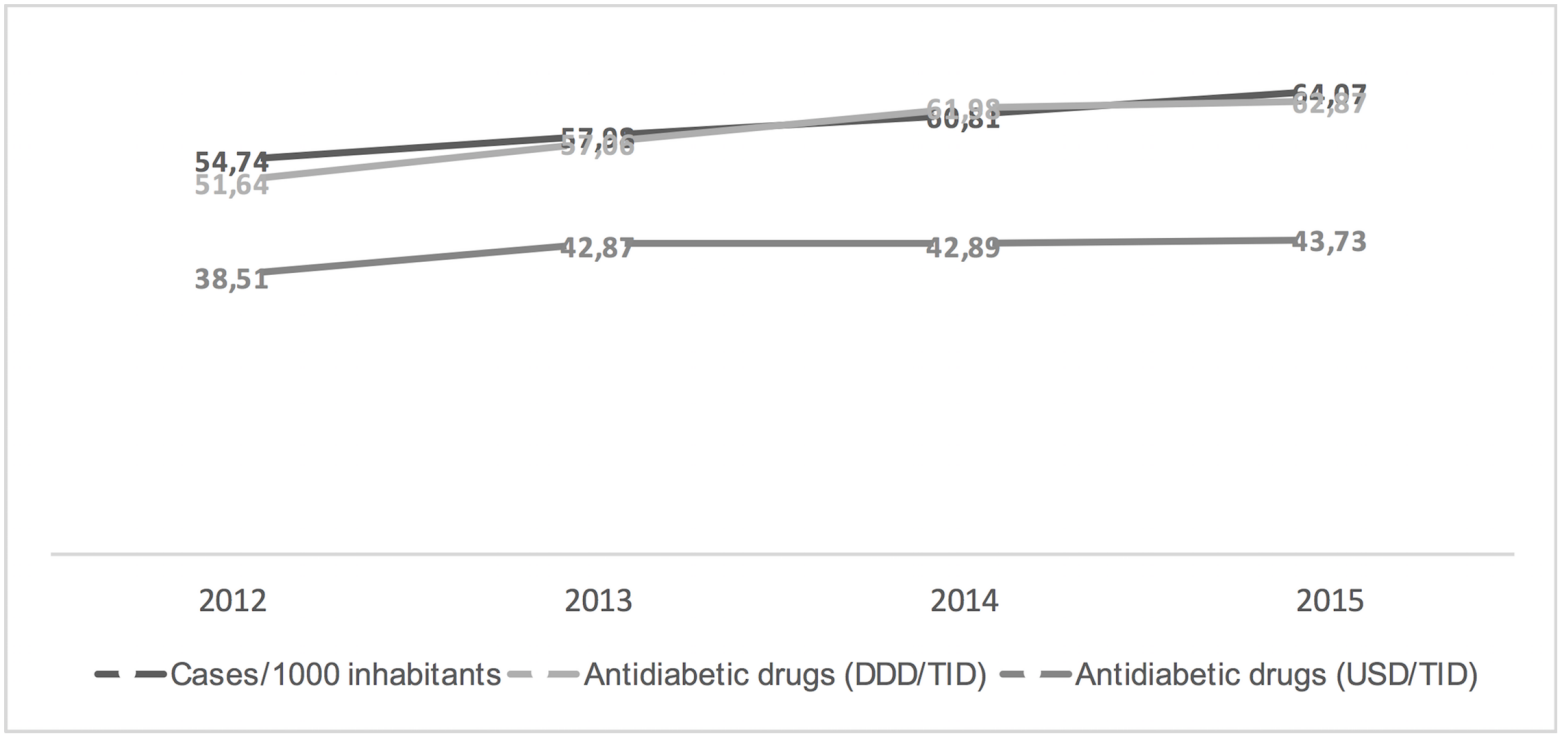
Trends in the diabetes mellitus morbidity (cases per 1000 inhabitants), antidiabetic drugs consumption (DDD/TID) and costs (USD/TID) in Poland, 2012–2015.

**Fig 2:**
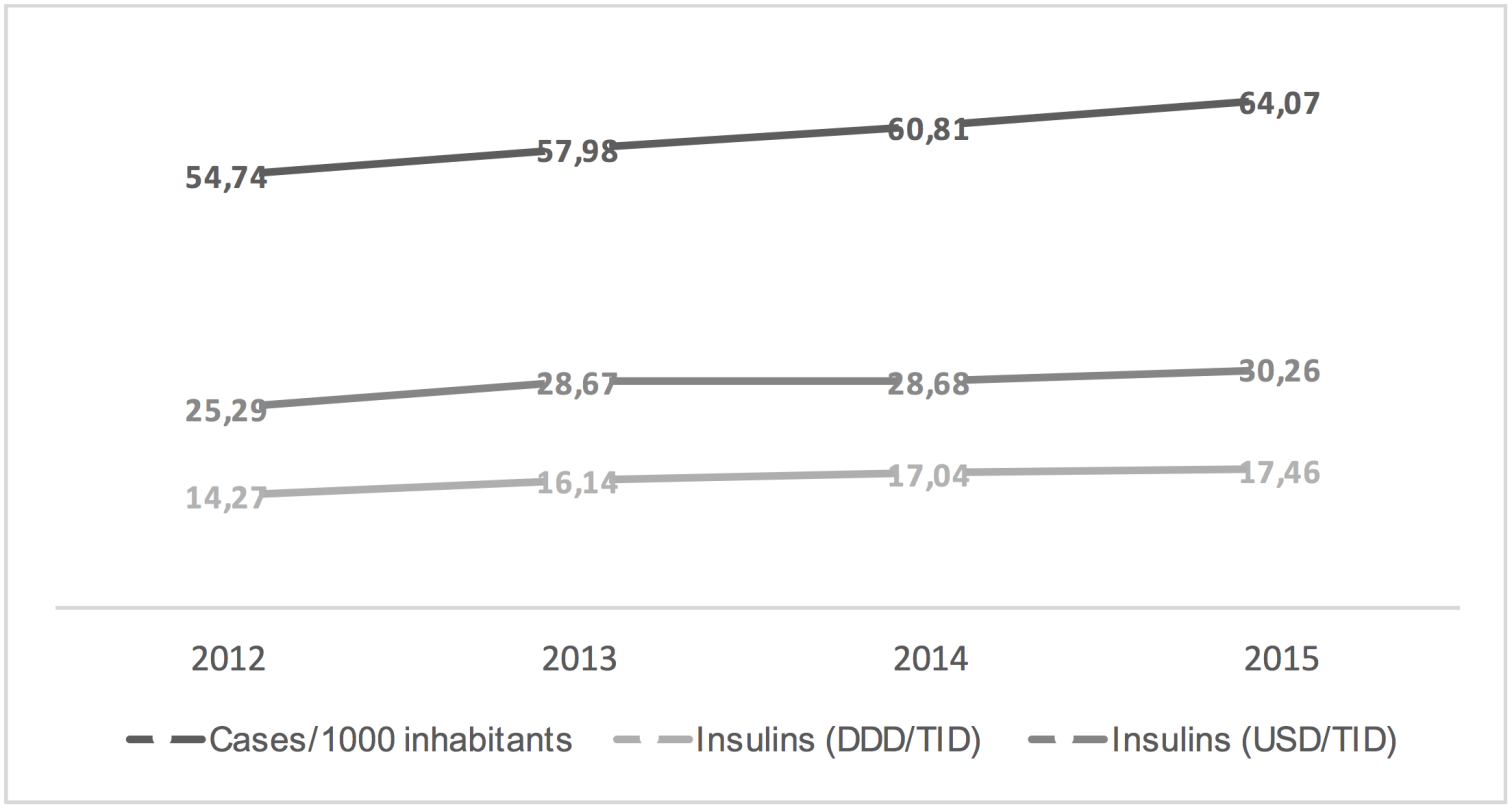
Trends in the diabetes mellitus morbidity (cases per 1000 inhabitants), insulin consumption (DDD/TID) and costs (USD/TID) in Poland, 2012–2015.

**Fig 3:**
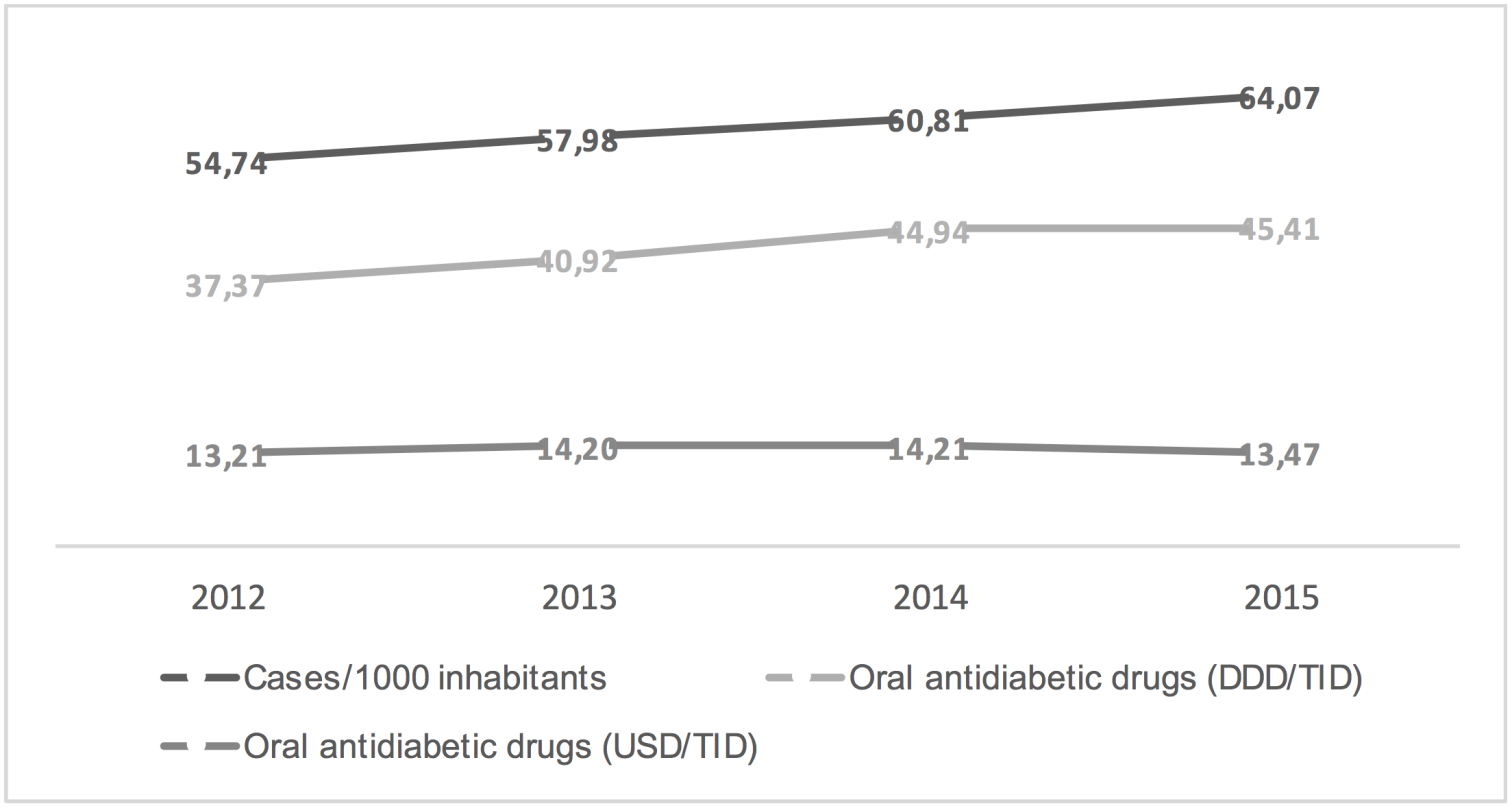
Trends in the diabetes mellitus morbidity (cases per 1000 inhabitants), oral antidiabetic drugs consumption (DDD/TID) and costs (USD/TID) in Poland, 2012–2015.

Figs 1–3 show the trends in the number of diabetes mellitus cases per 1000 inhabitants, consumption and costs of antidiabetics (total, insulins and oral drugs) over a study period. It is worthy of note that the cost of oral antidiabetic drugs was stable (13.21 USD/TID in 2012 and 13.47 USD/TID in 2015) while the utilisation rose from 37.37 DDD/TID in 2012 to 45.73 DDD/TID in 2015. This difference in utilization and cost trends can be attributed to reduction in prices of drugs and a restricted migration of patients toward newer more expensive drugs.

### Analysis of volume and costs of insulins

The changes of volume and costs of insulins were analysed by grouping the products according to their international name (Table 2), duration of action and molecular origin (Table 3). Consumption of both human insulins and insulin analogues increased and an accompanying cost increase was noticed. However, a 14% increase of issued human insulin DDDs corresponded to a 10% increase of the costs, while a 17% increase of issued insulin analogues DDDs corresponded to a 38% increase of costs. Taking into consideration the number of DDDs, human insulins are still dominating (127,013,000 in 2012 and 145,163,000 in 2015), but their share in the total volume has dropped from 63% to 59%. Biphasic human insulin share has declined, whereas the share of fast-acting insulin analogues, long-acting human insulins and long-acting insulin analogues has risen (Table 3). The higher increase (by 288%) was noticed in the class of long-acting insulin analogues (glargine, detemir), but their share in the insulin volume remains the smallest (4%) (Table 3). The rate of expansion of long-acting human insulins and fast-acting analogues volume has reached the values of 53% and 30% respectively, whereas for biphasic insulins—only 5% (Table 3).

**Table 2:** Volume and cost of insulins by insulin type in Poland. Number of DDDs in thousands. Costs in USD x 1000.

Insulin type		2012	2013	2014	2015
Biphasic isophane insulin	Volume; DDD (%)	73,313 (36.43)	78,975 (34.91)	79,200 (33.1)	76,950 (31.41)
	Cost; USD (%)	114,835 (32.19)	122,291 (30.36)	120,801 (28.54	117,125 (26.82)
Biphasic insulin lispro	Volume; DDD (%)	12,525 (6.22)	13,200 (5,83)	13,613 (5.69)	13,125 (5.36)
	Cost; USD (%)	25,593 (7.17)	26,743 (6.64)	27,359 (6.44)	26,389 (6.04)
Neutral insulin	Volume; DDD (%)	23,325 (11.59)	26,100 (11.54)	21,038 (8.79)	21,750 (8.88)
	Cost; USD (%)	36,451 (10.22)	40,364 (10.02)	41,448 (9.76)	43,131 (9.88)
Insulin aspart	Volume; DDD (%)	46,313 (23.01)	52,013 (22.99)	54,225 (22.66)	55,613 (22.70)
	Cost; USD (%)	96,537 (27.06)	107,509 (26.69)	111,105 (26.17)	113,920 (26.09)
Insulin glulisine	Volume; DDD (%)	1,913 (0.95)	3,863 (1.71)	5,438 (2.27)	7,125 (2.91)
	Cost; USD (%)	3,676 (1.03)	7,357 (1.83)	10,316 (2.43)	13,465 (3.08)
Insulin lispro	Volume; DDD (%)	10,950 (5.44)	11,775 (5.20)	13,350 (5.58)	14,063 (5,74)
	Cost; USD (%)	22,441 (6.29)	23,849 (5.92)	26,807 (6.31)	28,274 (6.48)
Insulin isophanum	Volume; DDD (%)	30,300 (15.05)	34,688 (15.33)	44,100 (18.43)	46,463 (18.97)
	Cost; USD PPT (%)	47,535 (13.33)	53,818 (13.36)	56,251 (13.25)	59,068 (13.53)
Insulin zinc suspensio	Volume; DDD (%)	75 (0.04)	38 (0,02)	0 (0.00)	0 (0.00)
	Cost; USD (%)	41 (0.01)	37 (0.01)	0 (0.00)	0 (0.00)
Insulin detemir	Volume; DDD (%)	675 (0.34)	1,388 (0.61)	2,138 (0.89)	2,588 (1.06)
	Cost; USD (%)	2,648 (0.74)	5,423 (1.35)	7,994 (1.88)	9,529 (2.18)
Insulin glargine	Volume; DDD (%)	1,875 (0.93)	4,200 (1.86)	6,188 (2.59)	7,313 (2.98)
	Cost; USD (%)	6,950 (1.95)	15,428 (3.83)	22,521 (5.30)	25,744 (5.90)
Total	Volume; DDD (%)	201,263 (100.00)	226,238 (100.00)	239,513 (100.00)	244,988 (100.00)
	Cost; USD (%)	356,707 (100.00)	402,819 (100.00)	424,599 (100.00)	436,645 (100.00)

DDD: definded daily dose; USD: United States Dollar

**Table 3:** Volume and cost of insulins by class-duration of action and molecular origin in Poland. Number of DDDs in thousands. Costs in USD x 1000.

Insulin type		2012	2013	2014	2015
Biphasic/human	Volume; DDD (%)	73,313 (36.43)	78,975 (34.91)	79,200 (33.1)	76,950 (31.41)
	Cost; USD (%)	114,835 (32.19)	122,291 (30.36)	120,801 (28.54	117,125 (26.82)
Biphasic/analogue	Volume; DDD (%)	12,525 (6.22)	13,200 (5,83)	13,613 (5.69)	13,125 (5.36)
	Cost; USD (%)	25,593 (7.17)	26,743 (6.64)	27,359 (6.44)	26,389 (6.04)
Short/human	Volume; DDD (%)	23,325 (11.59)	26,100 (11.54)	21,038 (8.79)	21,750 (8.88)
	Cost; USD (%)	36,451 (10.22)	40,364 (10.02)	41,448 (9.76)	43,131 (9.88)
Short/analogue	Volume; DDD (%)	59,175 (29.40)	67,650 (29.90)	73,013 (30.51)	76,800 (31.35
	Cost; USD (%)	122,654 (34.39)	138,715 (34.44)	148,228 (34.91)	155,659 (35.65)
Long/human	Volume; DDD (%)	30,375 (15.09)	34,725 (15.35)	44,100 (18.43)	46,463 (18.97)
	Cost; USD (%)	47,576 (13.34)	53,855 (13.37)	56,251 (13.25)	59,068 (13.53)
Long/analogue	Volume; DDD (%)	2,550 (1.27)	5,588 (2.47)	8,325 (3.48)	9,900 (4.04)
	Cost; USD (%)	9,598 (2.69)	20,851 (5.18)	30,515 (7.19)	35,273 (8.08)
Total	Volume; DDD (%)	201,263 (100.00)	226,238 (100.00)	239,288 (100.00)	244,988 (100.00)
	Cost; USD (%)	356,707 (100)	402,819 (100)	424,599 (100)	436,645 (100)

DDD: definded daily dose; USD: United States Dollar

## Discussion

Our study provides an insight into the national trends in antidiabetic medication utilization and expenditures. It highlights the growing shift away from sulfonylureas towards the use of biguanides to treat diabetes mellitus. The high use of biguanides (metformin) and the decline in sulfonylureas and alpha glucosidase inhibitors indicates that the clinical practice in Poland is in line with the international ADA and EASD guidelines [8, 9]. These guidelines recommend metformin as a first-line therapy in type 2 diabetes due to its low cost, proven safety, weight neutrality and possible benefits on cardiovascular outcomes [9,10].

The use of DPP4 inhibitors in Poland is very low even though international guidelines recommend them as one of the alternatives in combination therapy with metformin, when metformin monotherapy proves ineffective [8, 9]. This group of drugs is not reimbursed in Poland due to insufficient information on their long-term use safety in clinical practice and particularly high therapy costs. Thiazolidinediones are not used in Poland which can be related to worse safety profile [11, 12], recommendation of cautionary use and/or availability of safer therapeutic options.

The insulin market is still dominated by human insulins although their share in volume slowly declines. The moderate increase in volume of biphasic insulin with a significant increase in volume of long-acting insulin (both human insulin and insulin analogues) and fast-acting analogues indicates that the basal-bolus is the basic insulin regimen. It should be emphasised that despite a very dynamic increase of the volume of long-acting insulin analogues, the dominating basal insulin is the insulin isophane (NPH). This is the result of restrictions of the reimbursement scheme of long-acting insulin analogues. They are reimbursed:

1. since 07/01/2012 in type 1 diabetes in adults, teenagers and children at least 2 years old (detemir) or at least 6 years old (glargine),
2. since 09/01/2013 in type 2 diabetes patients treated with NPH insulin for at least 6 months and with HbA1c ≥8%, in type 2 diabetes patients treated with NPH insulin for at least 6 months and with documented recurring episodes of severe or nocturnal hypoglycaemia, and in diabetes with a known cause (in accordance with the WHO definition)

and available for a payment of 30% (approx. USD 41.11 /package), and not a lump sum (USD 2.25)/package) as in the case of human insulin isophane (NPH).

From 09/01/2015 the cheaper, biosimilar insulin glargine is included in the reimbursement scheme, establishing a new lower financing limit in the long-acting insulin analogues group, which has reduced the patient’s co-payment by 26%. As economic aspects strongly influenced the prescribing patterns it is expected that in the coming years a higher proportion of diabetic subjects will be treated with long-acting analogues.

In Poland, the fast-acting insulins are dominated by analogues. They are reimbursed for a lump-sum payment with a limit set by fast-acting human insulins, which is why patient’s co-payment for a package varies from USD 18.37 (insulin glulisine) to USD 24.06 (insulin aspart).

The analysis of the insulin market shows that the use of analogues meets the newest ADA and EASD guidelines, which recommend using both NPH insulin and long-acting analogues as basal insulin [8, 9]. These guidelines point out, however, that the latter causes significantly less nocturnal hypoglycaemias (insulin glargine, insulin detemir) and slightly less body weight gain (insulin detemir), but is notably more expensive than NPH insulin. Due to a better pharmacokinetic profile compared to fast-acting human insulin, fast-acting analogues are recommended if fast-acting insulin injections before meals are required. The statement emphasises that an individual approach to the treatment of diabetes, that is establishing the treatment goal appropriate to the patient’s characteristics and selection of individual therapy, is necessary. It has been also stated that the cost may play an important role in drug selection: since the prices of new drugs are increasing, doctors should take into account the patient’s private and social resources, and if possible recommend appropriate use of cheaper generic products [9].

Publications assessing the diabetes medication market in other countries apply to different years and often refer to an earlier than analysed above period, which somewhat hinders the direct comparison of the results. Despite this, similarities in the consumption of some drugs were noticed, e.g. metformin is the most frequently prescribed diabetes medication, and consumption of sulfonylureas is diminishing [13–25]. The differences between Poland and other countries were observed in the adoption of new antidiabetic drugs, e.g. in 2012 the share of DPP-4 inhibitors in the diabetes medication market amounted to approx. 10% in US and UK [14, 15], and only 0.003% in Poland. Moreover, Polish market lacks GLP-1 analogues, which in the UK constitute to 1.1% [14], in the US to 1.6% [15] and in Denmark to 7% [18] of the prescribed diabetes medications.

In Poland human insulins are the most often prescribed type of insulin (59% in 2015), whereas in the US and UK the insulin market is dominated by insulin analogues (89% and 82% of prescribed insulins, respectively) [15, 26]. The market share of long-acting, fast-acting and biphasic analogues was respectively 52%, 27% and 8% in the US in 2012 [23] and 26.3%, 27.1% and 22.7% in the UK in 2008 [13]. At the same time the share of human NPH insulin decreased to 4% in the US and 5.6% in the UK. The increase of the share of insulin analogues certainly resulted in the increase of costs, however there is no evidence whether it contributed to improving the treatment outcomes [27].

The scientific evidence analysis [28] indicates that the benefits of using long-acting analogues over NPH insulin are moderate. Although their use results in a lower body weight gain (approx. 1 kg), decrease the risk of nocturnal hypoglycaemia and provide a more convenient regimen, the clinical significance of these advantages remains unclear. With no evidence that the long-acting insulin analogues improve safety, control glycaemia and reduce the risk of long-term complications of diabetes, NICE recommends caution in prescribing this group of drugs. The NICE guidelines, published in 2009 and updated in 2015 [29], recommend human insulin NPH as a drug of first choice in patients who require insulin therapy and introduce restrictions on indications for the use of long-acting analogues. The long-acting analogues are an alternative for insulin NPH in patients who: 1) need help with injecting insulin and for whom the use of long-acting analogues will reduce the frequency of insulin injections from 2 to 1 per day, 2) patient’s activity is reduced by recurring symptomatic hypoglycaemia symptoms, 3) an alternative for the analogue is 2 injections of insulin NPH combined with oral diabetes medication [8, 9]. Patients may be switched from insulin NPH to long-acting analogues if the intended level of HbA1c could not be reached due to hypoglycaemia episodes, or hypoglycaemias are occurring regardless of HbA1c. The change will result in them being able to perform the injections on their own or the number of injections will be reduced [29]. However, the increased consumption of long-acting analogues in the UK indicates that NICE recommendations are not fully implemented in practice. Whereas in Poland, long-acting analogues are being used among patients for whom they are the most beneficial thanks to the HTA Agency recommendation of restricting indications and a reference price system.

In summary, our study indicates that the observed tendencies, e.g. the increased consumption of metformin and the restricted use of long-acting insulin analogues, correspond to the evidence-based HTA recommendations. The reimbursement status has a deciding impact on how a drug is used, e.g. non-refunded incretin mimetics are practically absent from the segment financed by public resources and the increase of the consumption of long-acting analogues occurred only in year 2012/13, when these drugs were included in the reimbursement scheme. Ambiguous data on the long-term safety of new drugs significantly delay the decision of reimbursement, as now seen in the case of incretin mimetic drugs and as previously happened with the long-acting analogues. The cost of the drugs, or to be more precise, the ratio of cost to clinical outcomes, is an important factor as well. The uncontrolled growth of costs may only be stopped with rigorous guidelines optimising the treatment of diabetes being applied in practice.

The strength of our study is that it is based on data from the entire Polish population. A limitation of our study is that in our analysis of trends in prescribed anti-diabetic medication we do not distinguish between the different types of diabetes. Using routinely collected data does not allow us to make distinction between type 1 and type 2 diabetes. Since we extracted data on the number of patients receiving at least one prescription of any antidiabetic medication, some diabetic patients may have been misclassified. Our study applies to the consumption and costs of blood glucose-lowering therapies; however it does not analyse the consumption and costs of drugs used in relation to diabetes complications. Like other administrative database based studies it does not contain information on biochemical parameters, making it impossible to directly assess the impact of the use of various therapeutic classes on health outcomes. Despite this limitation, the results of our study provide useful insights into current prescribing practices and could serve policy makers as the basis for drug policy planning.
